# Exploring the Effects of Charge Modulation on the Reaction Energetics of the Oxygen Evolution Reaction in BiVO_4_‑Based Photocatalysts

**DOI:** 10.1021/acsomega.5c04810

**Published:** 2025-10-13

**Authors:** Hongjiang Chen, Patrick H.-L. Sit

**Affiliations:** † School of Energy and Environment, 53025 City University of Hong Kong, Tat Chee Avenue, Kowloon 999077, Hong Kong, China; ‡ City University of Hong Kong Shenzhen Research Institute, Shenzhen 518057, China

## Abstract

Photocatalytic water splitting is a promising and green approach for solar energy storage and hydrogen production. However, its efficiency is limited by the sluggish oxygen evolution reaction (OER), which requires high overpotentials. Bismuth vanadate (BiVO_4_) has gained significant attention as a semiconductor photocatalyst due to its favorable properties, including a narrow band gap and chemical stability. Nevertheless, the photoelectric conversion efficiency of the material remains low, and the slow water oxidation kinetics is considered one of the main causes. In this study, density functional theory calculations are performed to investigate the factors that influence the reaction energetics of the OER process on the BiVO_4_ surface. Our results reveal a strong correlation between the excess charge provided to BiVO_4_ and the reaction energy of the OER steps. Notably, negative charges increase the second and fourth reaction energy, while positive charges have the opposite effect. This behavior is consistent across different modification methods, such as introducing vacancies, doping other elements, and forming heterojunctions. The findings highlight the potential for tuning the reaction energy and overpotential of BiVO_4_-based photocatalysts through strategic modifications, advancing their application in efficient solar-driven water splitting.

## Graphical abstract

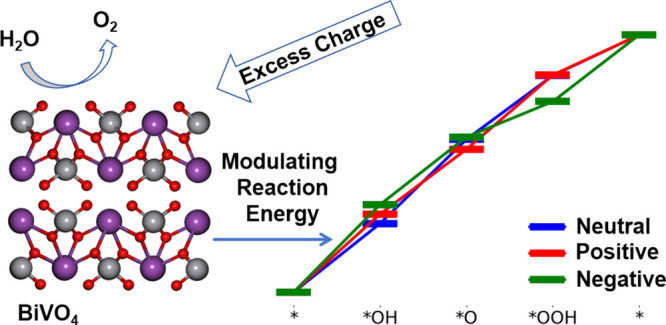

## 1 Introduction

To address the escalating threats of global warming, the demand for renewable energy is expected to surge rapidly in the coming decades. Among various environmentally friendly energy sources, solar energy stands out due to its vast availability and potential for large-scale application.^1^ However, the practical utilization and storage of solar energy face significant challenges. The inherent intermittency of solar power—due to its dependence on weather conditions and daylight—can disrupt the energy supply, while its low energy density necessitates advanced storage solutions. These limitations require the development of efficient energy storage systems and innovative technologies to capture, store, and deploy solar energy effectively, ensuring a reliable and continuous energy supply. Since the breakthrough discovery of the photocatalytic performance of TiO_2_ by Honda and Fujishima in 1972,^2^ photoelectrochemical (PEC) technology has emerged as one of the most promising solutions for solar energy storage. PEC technology enables the conversion of solar energy into hydrogen fuel through water splitting, which offers a clean and sustainable method for storing solar power. Hydrogen, produced via this method, can be stored and later utilized as a high-energy-density fuel, providing a flexible and scalable solution to the intermittent issue of solar energy.

Photocatalytic water splitting involves two half-reactions: the hydrogen evolution reaction (HER) at the cathode and the oxygen evolution reaction (OER) at the anode. While both reactions are essential for complete water splitting, it is typically the OER that imposes greater constraints on the system performance. The OER is a multistep process involving a four-electron transfer, making it a kinetically sluggish reaction. As a result, high overpotentials are needed to reach the required current densities for efficient water splitting. In practical electrolytes, the input potential often surpasses the theoretical value of 1.23 eV.

To realize highly efficient photocatalytic water splitting, it is imperative to enhance the OER activity. Among various semiconductor photocatalysts, bismuth vanadate (BiVO_4_) has garnered widespread attention due to its favorable properties that make it a promising candidate for high-performance photocatalysis.^3^ BiVO_4_ offers several advantages, including chemical and thermal stability and nontoxicity. A narrow band gap of approximately 2.4 eV also enables it to efficiently absorb visible light, a critical requirement for solar-driven water splitting. Additionally, BiVO_4_ possesses a favorable valence band (VB) position (with an EVB ≈ 2.4 V vs RHE), making it suitable for driving the OER. This valence band position is sufficiently positive to provide the thermodynamic driving force for water oxidation, which is essential for efficient oxygen evolution. A combination of these properties allows BiVO_4_ to effectively utilize sunlight to produce charge carriers (electrons and holes) that can participate in the water-splitting process.^4^

However, despite its potential, the practical application of BiVO_4_ is often limited by factors such as its poor charge transport ability,^5^ rapid electron–hole recombination,^6,7^ and relatively low surface reactivity.^8,9^ To overcome these limitations, significant efforts have been made to improve the performance of the BiVO_4_ photocatalysts. Strategies such as doping, crystal plane engineering, morphology control, macroporous/mesoporous structures, and the construction of heterojunctions have been explored to enhance its charge separation efficiency and catalytic activity.^10–12^

Remarkably, coupling BiVO_4_ with another semiconductor material to construct a heterojunction is an important way to improve the performance. Many studies have proven that the charge separation and transfer of the photoanode can be affected by the material properties used to construct the heterojunction and the interface formed in heterojunctions. Among these heterojunctions, BiVO_4_/WO_3_, BiVO_4_/ZnO_2_, and BiVO_4_/TiO_2_ have been synthesized experimentally and shown good performance.^13–18^ Nanorod array WO_3_/BiVO_4_ heterojunction films have a high incident photon-to-current efficiency, and a maximum photocurrent density of ∼5.35 ± 0.15 mA/cm^2^ is achieved at 1.23 V (vs RHE).^13,18^ The BiVO_4_/ZnO quantum dot heterostructure showed the greatest photocurrent density (5.5 mA/cm^2^ at 1.23 V vs RHE) in the absence of a cocatalyst and exhibited the lowest photoluminescence spectroscopy intensity compared to those of bare ZnO and BiVO_4_ ^17,19^ For BiVO_4_/TiO_2_, the incident photon-to-current efficiency is higher than that of single TiO_2_ or BiVO_4_, and 2.2 mA/cm^2^ current density at 1.23 V_RHE_ under visible light irradiation was obtained.^15,16^

Besides, defect engineering also achieved some progress.^20^ A record charge separation efficiency of 98.2% was found on a planar BiVO_4_ photoanodes with in situ formatted oxygen vacancies, and the light utilization at 400–520 nm was found to be increased.^21,22^ Li et al. recently achieves a high photocurrent density of 6.34 mA cm^–2^ via gradient distribution of oxygen vacancies.^23^ Tayyebi et al. reported that the photoelectrochemical performance of BiVO_4_ with bismuth vacancies showed an enhanced and stable photocurrent density of 1.2 mA/cm^2^ at 1.0 V vs Ag/AgCl as compared with a negligible photoresponse (15 μA/cm^2^) of tetrahedron phase BiVO_4_.^24^ A minor fluorescence peak was observed in the region between 610 and 650 nm for the vanadium vacancy BiVO_4_ photoanode, although only half or less photocurrent densities were obtained compared with a stoichiometric ratio BiVO_4_.^25^

Furthermore, doping also results in an improvement in the PEC performance of BiVO_4_.^26^ For example, a new absorption band with a 1.84 eV energy threshold appears in the Cr-doped BiVO_4_,^27^ and the absorption edge was found to extend from 499 to 505 nm in the Ti-doped BiVO_4_.^28^ In Mo-doped BiVO_4_, the charge separation efficiency exceeds 90% at 420 nm.^29^

Novel BiVO_4_-based photocatalysts with better performance keep emerging, but the complicated OER process makes theoretical research a tough task. Through density functional theory (DFT) calculations, the BiVO_4_(010) facet is confirmed to be more stable and energetically favorable to O_2_ evolution by Yang et al. and Bhatt et al.^30,31^ The electronic and optical properties of black phosphorus/BiVO_4_ heterojunction were investigated by Chen et al, showing that optical absorption was increased and an internal electric field was formed.^32^ Hu et al. studied the OER process on defected BiVO_4_ with oxygen vacancies, suggesting a more active vanadium site for PEC water splitting on an O_vac_ BiVO_4_ surface.^33^ Recently, excess charge was found to enhance the water adsorption on Mo-doped BiVO_4_ by Wang et al.,^34^ and the formation of localized polarons resulting from excess charge is discussed.^35–37^ Localized excess electrons is also found in Ca-intercalated g-C_3_N_4_, which leads to a more efficient reactive oxygen species (ROS) generation and reactant activation.^38^ Furthermore, another OER mechanism has been proposed in novel catalysts such as NiFeOx.^39,40^ Such a mechanism suggests the formation of O_2_ at an earlier step. Gono et al. performed an in-depth theoretical study with this pathway on several materials, including TiO_2_, RuO_2_, IrO_2_, Ni_2_P, and BiVO_4_, and found that the linear scaling relationship is broken and the overpotential may be reduced through this mechanism.^41^ Previous studies have been conducted on the reaction energy of different novel BiVO_4_-based materials,^42,43^ but the role of excess charge in the reaction energy was not clearly identified. Such electron richness/deficiency systems need in-depth exploration.

In this article, we conduct a detailed study on the reaction energies of the OER steps on different BiVO_4_-based systems using DFT calculations. To better understand how the reaction energy of the OER steps on BiVO_4_ surfaces can be affected, the following systems were studied: (1) pristine models of neutral BiVO_4_ and the models with one positive or negative charge; (2) vacancy model with a Bi vacancy, an O vacancy, or a V vacancy; (3) Ti-doped and Cr-doped model with the dopant replacing V; (4) heterojunctions of BiVO_4_ with WO_3_, ZnO_2_, and TiO_2_. Our calculations suggest that the reaction energy is affected by the net excess charge on BiVO_4_, which was confirmed in all of the studied systems. To identify the underlying reason that reaction energies vary among different systems, the binding energy between the −OH/–O group and the BiVO_4_ surface are analyzed. Furthermore, O_2_ formation is also found to be affected by electron richness/deficiency via an alternative pathway.

## 2 Computational Details

DFT calculations with plane-wave basis sets were performed with the PWscf module of the Quantum Espresso software package.^44^ Ultrasoft pseudopotentials with the Perdew–Burke–Ernzerhof exchange correlation functional were used.^45,46^ The kinetic energy cutoff is 50 Ry for the wave functions and 650 Ry for the augmented electronic density. The bulk structure of BiVO_4_ has the unit cell dimension of 5.19 × 11.70 × 5.09 Å^3^, and α = γ = 90°, β=90.38°, which is in accordance with the experiment.^47^ Then, the (010) surface with the 2 × 2 × 1 supercell was constructed with a 20 Å vacuum space introduced in the *z*-direction. The final slab of BiVO_4_ has 12 atomic layers and 96 atoms. The gamma angle was adjusted from 90.38° to 90° to fit the heterojunction better. The simulation cell has the dimensions of 10.17 × 10.38 × 32.01 Å^3^, α = γ = β = 90°. Structural optimization calculations were performed with all of the atoms that were free to move while the cell volume was fixed.

The four reaction steps of the OER considered are as follows:

$$M+H_{2}O\to M-\mathrm{OH}+H^{+}+e^{-}$$ 1

$$M-\mathrm{OH}\to M-O+H^{+}+e^{-}$$ 2

$$M-O+H_{2}O+e^{-}\to M-\mathrm{OOH}+H^{+}+e^{-}$$ 3

$$M-\mathrm{OOH}\to M+O_{2}+H^{+}+e^{-}$$ 4

Here, M represents the catalytic BiVO_4_ surface (pristine or modified).

Since *E*(H^+^ + e−) = *E*(1/2H_2_), the reaction energy in our study is calculated as below:

$$\Delta E_{1}=E(M-\mathrm{OH})-E(M)+1/2E(H_{2})-E(H_{2}O)$$ 5

$$\Delta E_{2}=E(M-O)-E(M-\mathrm{OH})+1/2E(H_{2})$$ 6

$$\Delta E_{3}=E(M-\mathrm{OOH})-E(M-O)+1/2E(H_{2})-E(H_{2}O)$$ 7

$$\Delta E_{4}=E(M)-E(M-\mathrm{OOH})+1/2E(H_{2})+E(O_{2})$$ 8

where *E*(M), *E*(M–O), etc., are the DFT energies for each species.

To avoid the direct effects of the defects/dopants on the OER reaction site, the vacancies of Bi and V and the dopants, Cr and Ti, were placed with several layers separated from the surface. To construct the heterojunction of BiVO_4_/WO_3_, BiVO_4_/ZnO, and BiVO_4_/TiO_2_, bulk structures of WO_3_,^48^ ZnO_2_,^49^ and TiO_2_ ^50^ were obtained from the experimental data. Each structure was adjusted to match the 2 × 2 × 1 BiVO_4_ slab as much as possible. Details of the structures of the systems studied and the location of vacancies and dopants are shown in Figure S1 in the Supporting Information (SI). The details of building heterojunctions can be found in Figure S2 in the SI. The 2 × 2 × 1 *k*-point mesh was used to sample the Brillouin zone of these metal surfaces by the Monkhorst–Pack method in the calculations.^51^ The Grimme-D2 scheme was used for the van der Waals correction.^52,53^

The Bader charge^54^ was used to estimate the excess charge. For doped systems, the excess charge was defined as the difference of the Bader charge between the doped atom and V. For vacancy systems, the excess charge was defined as the difference between the charge of a neutral atom and the charge of that atom in BiVO_4_. For heterojunction systems, the excess charge was defined as the charge transferred to BiVO_4_ from the heterojunction compound.

## 3 Results and Discussion

### 3.1 OER Process on Pristine BiVO_4_

The reaction energies and intermediate structures for the OER process on pristine BiVO_4_ are shown in Figure 1. Some previous studies showed that two active sites (Bi and V) are involved in the OER process on BiVO_4_,^30,33^ but other studies suggested that only Bi is involved as V tended to remain in a saturated VO_4_ structure.^55^ In view of this, in this study, different starting configurations were considered for each intermediate structure. Different possible intermediate structures and their relative energies are shown in Figure S3 in the SI. For M–OH, the −OH group was set to close to either the Bi atom or the V atom. We found that, no matter what the starting configuration, the final structure of Bi–OH for M–OH was obtained. On the other hand, for M–O, a metastable Bi–O structure was obtained, but the V–O structure has a lower energy. However, for M–OOH, two structures have been reported.^31^ The first structure is a double-site Structure A with the −OH group on Bi and the −O group on V. The other one is a single-site Structure B with the −OOH group on Bi, as shown in Figure 2. Our calculations showed that the energy of Structure B is only 0.03 eV lower than that of Structure A, which indicates that the two intermediate structures can coexist (also see the energy of Structures A and B in Figure S3d,e in the SI). However, in most of our other systems studied below, Structure A is more favorable. Therefore, in the following study, we focus on Structure A, and the notion for M–OOH indicates the two active site structures where the −OH group bonded with Bi and the −O bonded with V. The formation of an O–O bond from Structure A is expected to be the next step. The possible mechanisms were discussed in previous studies. Hu et.al. suggested a metastable structure where the two O from water form an O–O bond.^33^ On the other hand, Yang et.al. suggested the formation of superoxo intermediates, where the two O from water form a bridge with the surface O from BiVO_4_.^30^ After that, O_2_ is formed and detached from the surface.

**Figure 1:**
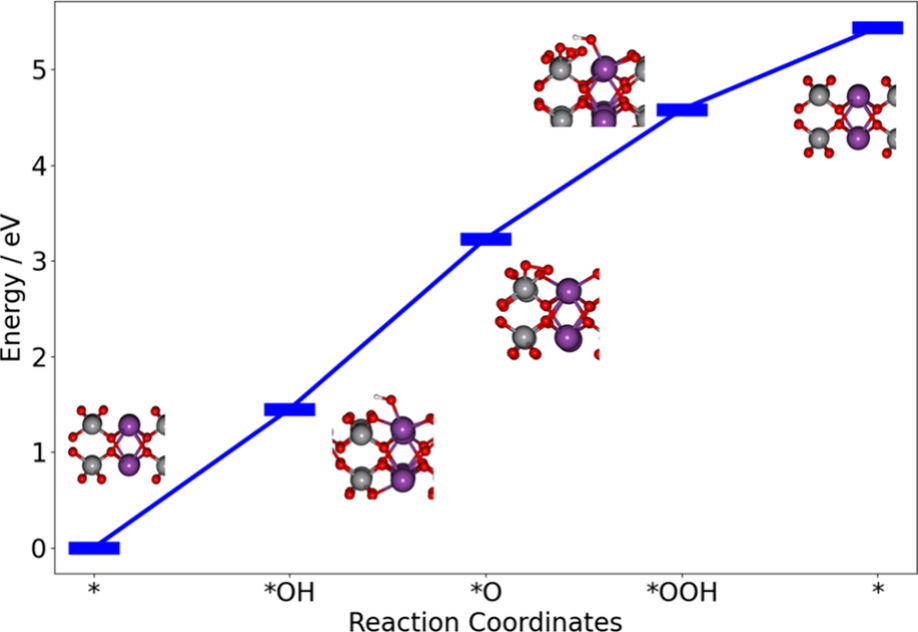
Reaction energies of the OER on the pristine BiVO_4_ (010) surface.

**Figure 2:**
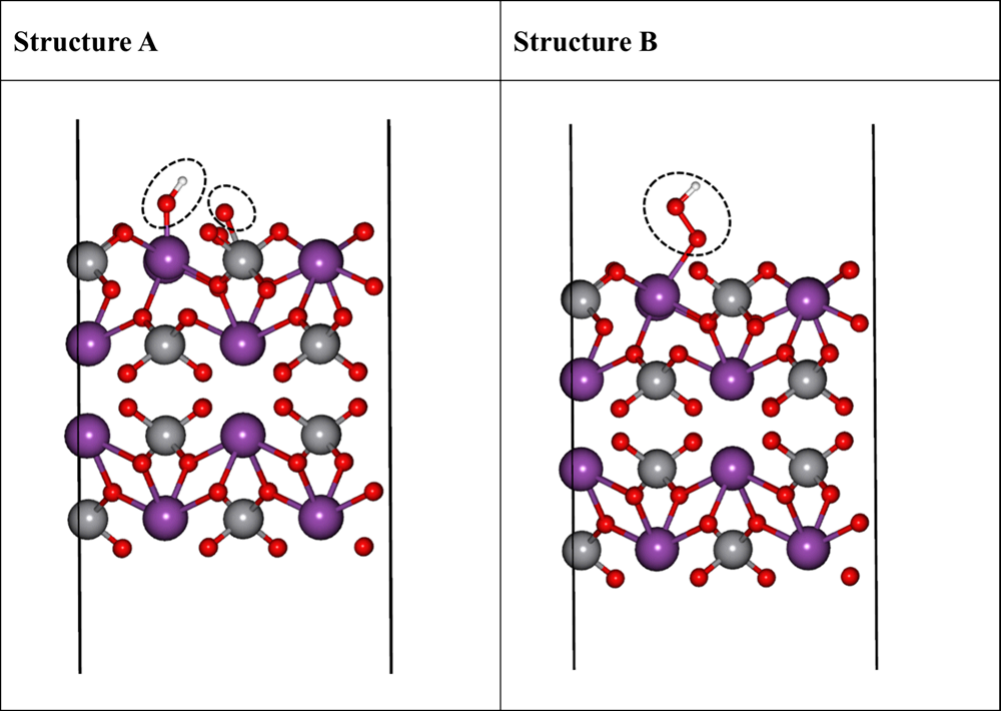
Two different structures of the M–OOH intermediates in the OER process and their energies. Structure A: the −OH group binds to Bi and the −O group binds on V. Structure B: the −OOH group binds on Bi. Species in the dashed ovals are the adsorbed species.

As a result, the overall process of the OER on the pristine BiVO_4_ can be described as follows: Step 1: One H_2_O adsorbs on the surface and loses one H. The resulting OH group bonds to Bi. Step 2: After losing another hydrogen, the oxygen from the water molecule switches to the nearby V. Step 3: Bi is ready to adsorb a second water molecule after the O switching after losing a H, where the −OH group from the second water bonds with Bi and the O still bonds with V. Step 4: Finally, after losing the last H, two adsorbed atoms combine to form O_2_ and then leave the surface. Steps 1–4 refer to the electron-transfer steps in eqs 1–4, respectively. The reaction energies for each step, shown in Table 1, are 1.45, 1.78, 1.34, and 0.86 eV, respectively.

### 3.2 Impact of Excess Charge on OER Step Energies

Here, we performed a comparative analysis of charged surfaces (±1 e), vacancy systems (Bi, V, O), doped systems (Cr, Ti), and heterojunction systems (BiVO_4_/WO_3_, BiVO_4_/TiO_2_, BiVO_4_/ZnO). We found that the interplay between the excess charges and the oxygen evolution energetics displays a remarkable influence across diverse BiVO_4_ modifications. Our results reveal that the electron-deficient systems (positive net excess charge on BiVO_4_) have higher Δ*E*_1_ and Δ*E*_3_ reaction energies but lower Δ*E*_2_ and Δ*E*_4_ reaction energies in cases of pristine, doped, and defect BiVO_4_. Conversely, the electron-rich environments (negative net excess charge on BiVO_4_) exhibit inverted trends, decreasing the reaction energy of Δ*E*_1_ and Δ*E*_3_ but increasing the reaction energies of Δ*E*_2_ and Δ*E*_4_ in such systems. This regulation is also partly followed when excess charge is introduced by forming heterojunctions. Compared to pristine BiVO_4_, all three heterojunction systems have a lower Δ*E*_1_ and Δ*E*_3_ and higher Δ*E*_2_ and Δ*E*_4_, with the Δ*E*_2_ of BiVO_4_/WO_3_ being very close to that of pristine BiVO_4_. However, across the heterojunction systems, electron-deficient BiVO_4_/WO_3_ has a higher Δ*E*_1_ and Δ*E*_3,_ lower Δ*E*_2_, and comparable Δ*E*_4_ compared to the electron-rich BiVO_4_/ZnO. The observed energy landscape bifurcation establishes a fundamental framework that connects charge control strategies with targeted OER step optimization. Figure 3 shows the general trend of Δ*E* compared between different electron-rich/deficient systems and pristine BiVO_4_, and detailed discussions for each system are provided in the following sections.

**Figure 3:**
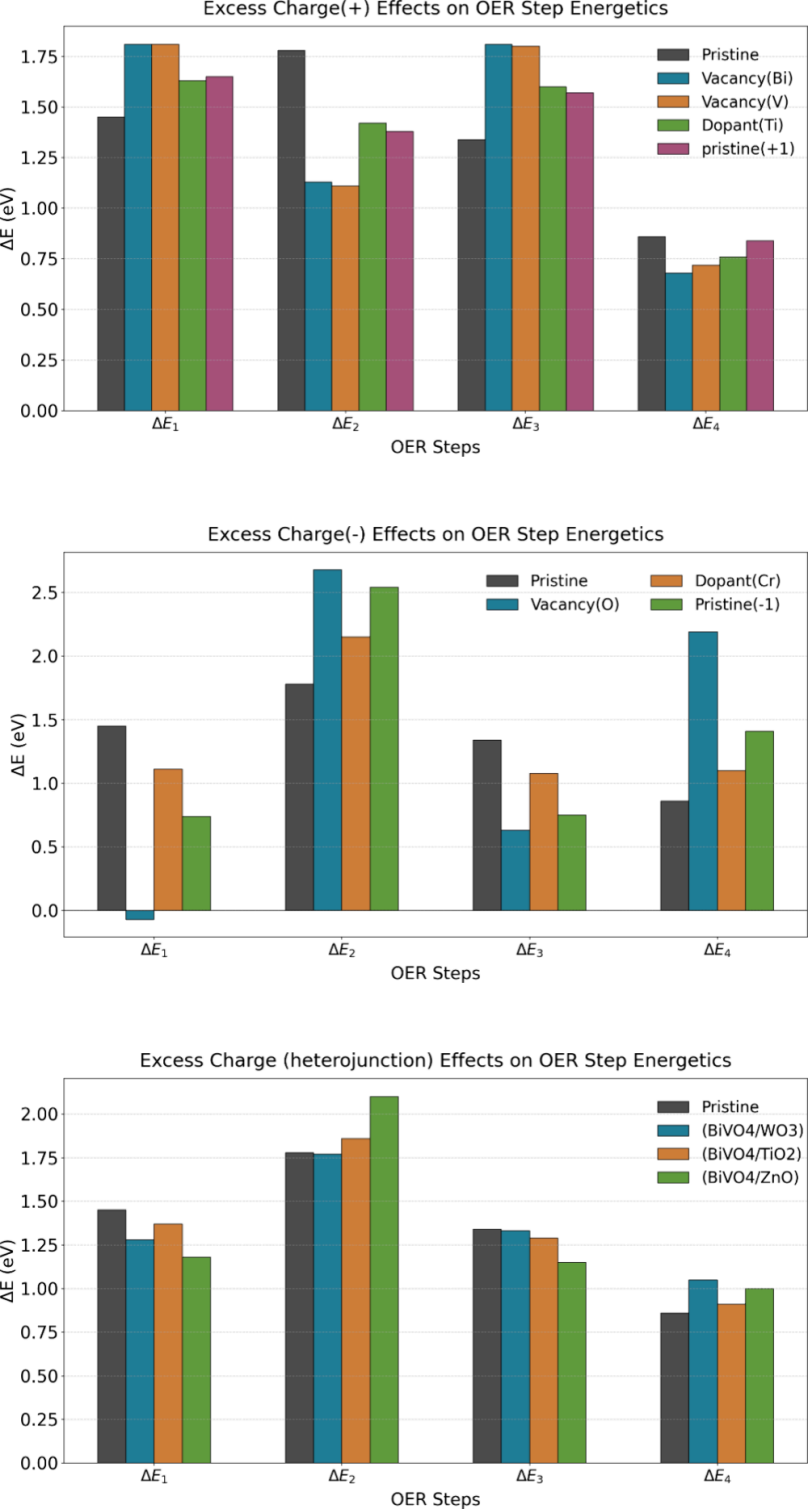
Reaction energetics of the OER steps of different electron-rich/deficient systems and pristine BiVO_4_.

#### 3.2.1 OER Process on Pristine BiVO_4_ with Artificial Extra Charge

We first set the total charge of a pristine BiVO_4_ to be ±1 to investigate the correlation between the excess charge and the OER energy on the BiVO_4_ surfaces. The results (Table 1) demonstrate that the variations in reaction energy for these idealized models follow clear patterns. To be specific, in the +1 charge system, Δ*E*_1_ and Δ*E*_3_ increase slightly (+0.20 and +0.23 eV relative to neutral BiVO_4_), while Δ*E*_2_ and Δ*E*_4_ decrease by 0.40 and 0.02 eV, respectively. Conversely, in the −1 charge system, Δ*E*_2_ and Δ*E*_4_ increase significantly (+0.76 and +0.55 eV), whereas Δ*E*_1_ and Δ*E*_3_ decrease by 0.71 and 0.59 eV.

**Table 1:** Reaction Energy of Oxygen Evolution on Pristine BiVO_4_ and the Charged BiVO_4_ Surface (in eV)

reaction	neutral	+1 charge	–1 charge
M → MOH (Δ*E*_1_)	1.45	1.65	0.74
MOH → MO (Δ*E*_2_)	1.78	1.38	2.54
MO → MOOH (Δ*E*_3_)	1.34	1.57	0.75
MOOH → M (Δ*E*_4_)	0.86	0.84	1.41
excess charge^a^	0	+1.00	–1.00

a Excess charges were defined as the total charge set to the system.

#### 3.2.2 OER Process on Defect BiVO_4_ with O, Bi, or V Vacancies

Creating vacancies is another commonly used method to improve photocatalyst performance, which also provides additional free positive and negative charge to the system. For BiVO_4_, an O atom vacancy can be considered as providing excess electrons, while the Bi and V vacancies generate holes. While the precise quantification of the excess charge in vacancy systems is challenging, we here estimated it by the Bader charge analysis that one oxygen vacancy donates approximately −1.07 e, and the Bi and V vacancies provide roughly +2.02 and +2.17 e, respectively. As expected, a similar trend was observed in vacancy systems (Table 2). The oxygen vacancy (O_vac_) decreased Δ*E*_1_ to −0.07 eV and Δ*E*_3_ to 0.63 eV, while Δ*E*_2_ and Δ*E*_4_ are substantially increased by 0.90 and 1.33 eV to 2.68 and 2.19 eV, respectively. In contrast, Bi and V vacancies (Bi_vac_ and V_vac_) increased Δ*E*_1_and Δ*E*_3_, while they decreased Δ*E*_2_ and Δ*E*_4_. For the Bi vacancy, Δ*E*_1_ and Δ*E*_3_ both changed to 1.81 eV; meanwhile, the change of Δ*E*_2_ to 1.13 eV and Δ*E*_4_ to 0.68 eV was noted. For the V vacancy, Δ*E*_1_ and Δ*E*_3_ reached 1.80 and 1.81 eV, respectively, while Δ*E*_2_ and Δ*E*_4_ measured 1.11 and 0.72 eV. The observed Δ*E* variations closely resemble those in the ideal models when considering oxygen vacancy-induced negative charges and metal vacancy-induced positive charges.

**Table 2:** Reaction Energy of Oxygen Evolution on the Defect BiVO_4_ Surface, with O, Bi, or V Vacancies (in eV)

vacancy	O_vac_	Bi_vac_	V_vac_
M → MOH (Δ*E*_1_)	–0.07	1.81	1.81
MOH → MO (Δ*E*_2_)	2.68	1.13	1.11
MO → MOOH (Δ*E*_3_)	0.63	1.81	1.80
MOOH → M (Δ*E*_4_)	2.19	0.68	0.72
excess charge^a^	–1.07	+2.02	+2.17

a Excess charges were calculated as the charge of a neutral O (or Bi, V) minus the Bader charge of the O (or Bi, V) in BiVO_4_.

#### 3.2.3 OER Process on Cr-Doped BiVO_4_ and Ti-Doped BiVO_4_

Recent studies reported that the Cr-doped and Ti-doped BiVO_4_ exhibit enhanced photocatalytic performance due to improved water adsorption.^28,56^ Substituting V with Cr or Ti induces an electron-rich or electron-deficient system, as Cr has one more d-electron and Ti has one less than V. Charge modulation quantification was achieved similar to the case in the vacancy systems; Cr and Ti dopants are estimated to introduce net charges of approximately −1.12 and +1.13 e by Bader charge distribution, respectively. The OER energies for doped BiVO_4_ are summarized in Table 3. The marked differences in reaction energies between doped and pristine BiVO_4_ strongly suggest that electron-rich or electron-deficient states profoundly influence the energy of the OER energetics. The trends align with expectations: Cr doping reduces Δ*E*_1_ and Δ*E*_3_ to 1.11 and 1.08 eV, respectively, while it increases Δ*E*_2_ and Δ*E*_4_ by 0.37 and 0.24 eV compared to pristine BiVO_4_, to 2.15 and 1.10 eV, respectively. Conversely, Ti doping elevates Δ*E*_1_ to 1.63 eV and Δ*E*_3_ to 1.60 eV, while it lowers Δ*E*_2_ to 1.42 eV and Δ*E*_4_ to 0.76 eV.

**Table 3:** Reaction Energies of Oxygen Evolution on Pristine BiVO_4_ and the Doped BiVO_4_ Surface (in eV)

dopant	pristine	Cr-doped	Ti-doped
M → MOH (Δ*E*_1_)	1.45	1.11	1.63
MOH → MO (Δ*E*_2_)	1.78	2.15	1.42
MO → MOOH (Δ*E*_3_)	1.34	1.08	1.60
MOOH → M (Δ*E*_4_)	0.86	1.10	0.76
excess charge^a^	0	–1.12	+1.13

a Excess charges were calculated as the Bader charge of V minus the Bader charge of Cr (or Ti) in BiVO_4_.

#### 3.2.4 OER Process on Heterojunctions BiVO_4_/WO_3_, BiVO_4_/TiO_2_, and BiVO_4_/ZnO

In heterojunction systems, the combination of BiVO_4_ with WO_3_, TiO_2_, and ZnO exhibits differences in the OER energy changes compared with the previous BiVO_4_-based systems (Table 4). For example, Δ*E*_1_ and Δ*E*_3_ in heterojunctions are all lower than that in the pristine BiVO_4_. For Δ*E*_2_, the changes are different for the different cases. For BiVO_4_/WO_3_, it is slightly lower (1.77 vs 1.78 eV), while the other two are higher. On the other hand, the Δ*E*_4_ values for the three heterojunctions are 0.91 eV (BiVO_4_/TiO_2_), 1.05 eV (BiVO_4_/WO_3_), 1.00 eV (BiVO_4_/WO_3_), which are all higher than Δ*E*_4_ of the pristine BiVO_4_ (0.86 eV) regardless of the sign of the excess charge.

However, the excess charge transferred from the heterojunction compounds still plays a role, as it does in the other systems studied in this work. Considering BiVO_4_/WO_3_ (+0.60 e) and BiVO_4_/ZnO (−0.72 e), although both have lower Δ*E*_1_ values compared to the pristine system, the electron-deficient WO_3_ heterojunction reduces Δ*E*_1_ by only 0.17 eV (from 1.45 → 1.28 eV), while the electron-rich ZnO heterojunction reduces it by 0.27 eV (from 1.45 → 1.18 eV). A smaller decrease in Δ*E*_3_ can also be noticed in the electron-deficient WO_3_ heterojunction. However, such smaller decreases in Δ*E*_1_ and Δ*E*_3_ in electron-deficient system were compensated by a larger increase in Δ*E*_4_. For Δ*E*_2_, WO_3_ shows a slight decrease and ZnO shows an increase, similar to the behaviors in other electron-rich/deficient systems.

**Table 4:** Reaction Energies (eV) of the OER on the Heterojunctions of the BiVO_4_ Surface

heterojunction	BiVO_4_/TiO_2_	BiVO_4_/WO_3_	BiVO_4_/ZnO
M → MOH (Δ*E*_1_)	1.37	1.28	1.18
MOH → MO (Δ*E*_2_)	1.86	1.77	2.10
MO → MOOH (Δ*E*_3_)	1.29	1.33	1.15
MOOH → M (Δ*E*_4_)	0.91	1.05	1.00
excess charge^a^	0.00	+0.60	–0.72

a Excess charges were defined as the charge transferred to BiVO_4_ from the heterojunction compound; a negative sign indicates BiVO_4_ gaining electrons.

### 3.3 Analysis of Δ*E* Trends and Relationship with Adsorption Energies of O and OH

A pronounced inverse linear relationship emerges between the excess charge (*Q*) and the step 1 reaction energy (Δ*E*_1_), characterized by a correlation coefficient *R* ^2^ = 0.831 (Figure 4a). As the charge shifts from electron-deficient (+1.00 to +1.81 e) to electron-rich (−0.72 to −1.12 eV), Δ*E*_1_ decreases from ∼1.81 eV (Bi_vac_) to 0.74 eV (−1e BiVO_4_). Δ*E*_2_ exhibits a strong positive linear dependence on excess charge, decreasing from 2.68 eV (O_vac_) to 1.11 eV (V_vac_), with a correlation coefficient *R* ^2^ = 0.948.

**Figure 4:**
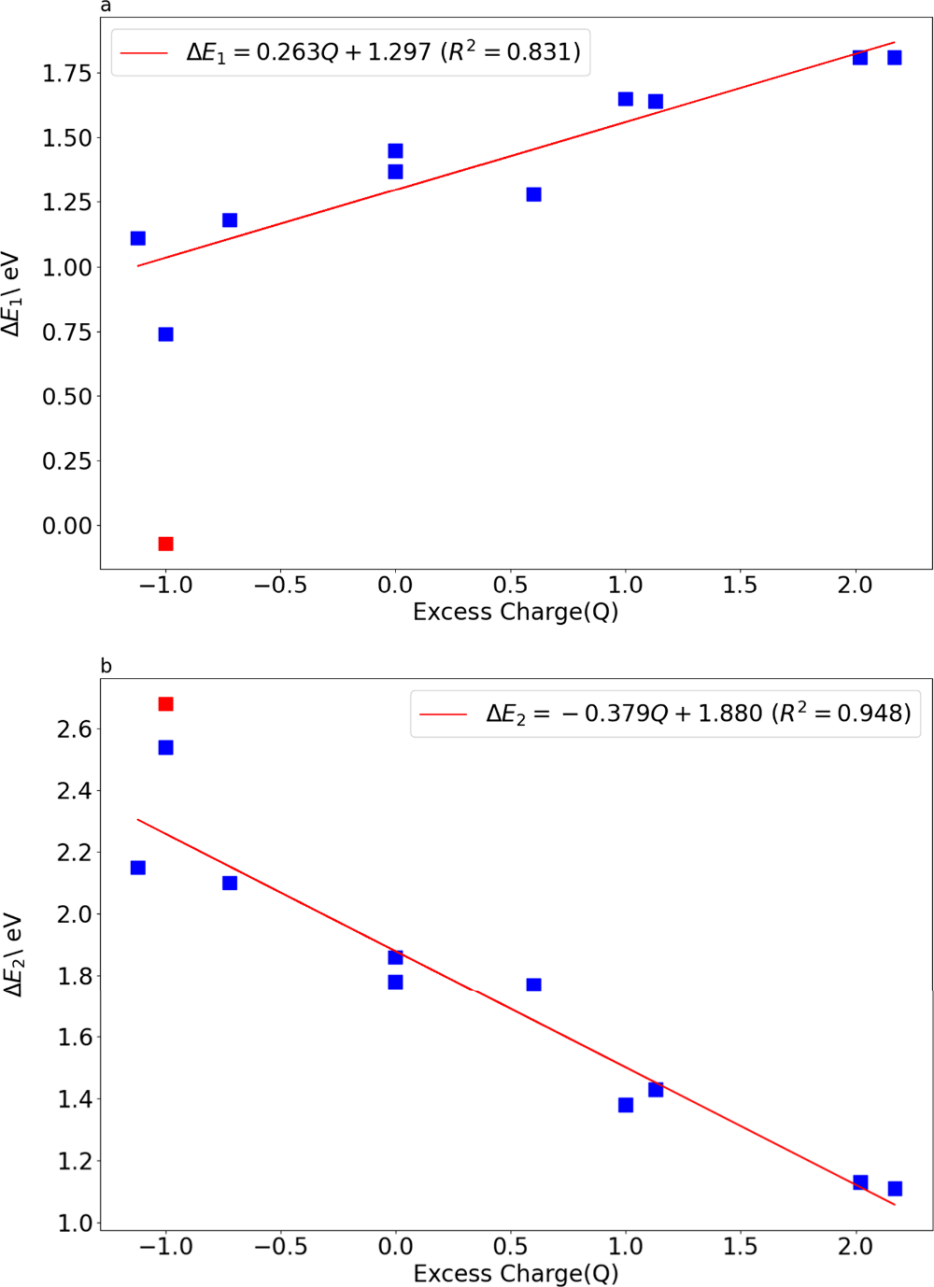
(a) Δ*E*_1_ and (b) Δ*E*_2_ vs excess charge. Data of O_vac_ (red dot) are not included for linear fitting (see linear fitting with the data of the O_vac_ in Figure S4 in the SI).

The following empirical relations were found:

$$\Delta E_{1}=0.263Q+1.297$$

$$\Delta E_{2}=-0.379Q+1.880$$

The O_vac_ system (charge *Q* = −1.07e) exhibits significant anomalies thus excluded in the linear fitting here, and Figure S4 in the SI shows the linear fitting including the O_vac_ system. According to the fitting relation Δ*E*_1_ = 0.263*Q* + 1.297, the predicted Δ*E*_1_ for O_vac_ is 1.01 eV, while the calculated value is −0.07 eV, resulting in a deviation of 0.94 eV. This value is well below the Δ*E*_1_ range of all other systems (0.74–1.81 eV) and is the only negative data point (Figure 4a). For Δ*E*_2_, the O_vac_ system also slightly deviates from the predicted value. Based on *Q* = −1.07e^–^, the fitted predicted Δ*E*_2_ is 2.28 eV, while the measured value is as high as 2.68 eV, resulting in a deviation of +0.40 eV. The origin of this deviation requires further investigation.

To further probe this relationship, we analyzed the adsorption energy (Δ*E*_ad_) of the key intermediates (M–OH and M–O). Table S1 presents the adsorption energies of M–OH (*E*_ad_ (M–OH)) and M–O (*E*_ad_ (M–O)) for each system, as well as their differences from pristine BiVO_4_ (Δ*E*_ad_ (M–OH) and Δ*E*_ad_ (M–O)).

By analyzing the changes of *E*_ad_, we can also reveal why there is a correlation between electron richness and the OER energy. Since the reaction energy can also be expressed as follows:

$$E_{\mathrm{ad}}(M-\mathrm{OH})=E(M-\mathrm{OH})-E(M)-E(\mathrm{OH})$$

$$E_{\mathrm{ad}}(M-O)=E(M-O)-E(M)-E(O)$$

Thus,

$$\begin{array}{ll} \Delta E_{1} & =E(M-\mathrm{OH})-E(M)+1/2E(H_{2})\\ & =E(M-\mathrm{OH})-E(M)-[E(\mathrm{OH}))+1/2E(H_{2})] \end{array}$$

$$\begin{array}{ll} \Delta E_{2} & =E(M-O)-E(M-\mathrm{OH})+1/2E(H_{2})\\ & =E_{\mathrm{ad}}(M-O)-E_{\mathrm{ad}}(M-\mathrm{OH})-[E(O)-E(\mathrm{OH}))+1/2E(H_{2})] \end{array}$$

The terms in the bracket are identical across different BiVO_4_-based systems. Therefore, the difference only arises from *E*_ad_(M–O) and *E*_ad_(M–OH).

The variation of ΔE_1_ and ΔE_2_ across different systems is fundamentally linked to the interplay between the M–OH and M–O adsorption strengths (*E*_ad_(M–OH) and *E*_ad_(M–O)). For ΔE_1_, which correlates directly with *E*_ad_(M–OH), a weakening of M–OH binding (i.e., less negative *E*_ad_(M–OH) relative to neutral systems) leads to an increase in ΔE_1_. Electron-deficient systems [Δ*E*_ad_(M–OH) = +0.19 to +0.36 eV] exemplify this trend, where the reduced adsorption strength elevates ΔE_1_ by 0.27–0.43 eV compared to the neutral value (1.38 eV). Conversely, in electron-rich systems [Δ*E*_ad_(M–OH) = −0.34 to −1.52 eV], the significantly enhanced M–OH binding drives ΔE_1_ downward, reaching as low as −0.07 eV. For ΔE_2_, governed by the differential between *E*_ad_(M–O) and *E*_ad_(M–OH), opposing trends emerge. In electron-deficient systems, enhanced M–O adsorption [Δ*E*_ad_(M–O) = −0.16 to −0.32 eV] combined with weakened M–OH binding amplifies the *E*_ad_(M–O)–*E*_ad_(M–OH) gap, causing Δ*E*_2_ to decrease by 0.36–0.58 eV relative to the neutral system (1.78 eV). In contrast, electron-rich systems exhibit diminished M–O adsorption gains [Δ*E*_ad_(M–O) = −0.62 to +0.05 eV], paired with a substantial strengthening of M–OH interactions, which narrows the *E*_ad_(M–O)–*E*_ad_(M–OH) difference. This synergistic effect elevates Δ*E*_2_ by up to +0.90 eV (e.g., Cr-doped system: Δ*E*_2_ = 2.10 eV). These results demonstrate that Δ*E*_1_ and Δ*E*_2_ exhibit antagonistic responses to charge-modulated adsorption energetics, with Δ*E*_1_ favoring weaker M–OH binding and Δ*E*_2_ requiring a balance of both M–OH and M–O interactions.

### 3.4 Impact of the Charge Modulation on the Reaction Path

Recently, another OER mechanism has been proposed to explain the deviations from linear scaling laws in some novel catalysts.^40,41^ Instead of eqs 3 and 4 in the last two steps as Path 1, the reaction can take place as the last two reaction steps, where the O_2_ was generated earlier at step 3 as Path 2. Equations 1–4 are the same as those shown in Section 2 but grouped into Path 1 and Path 2 of the reaction for easy reference. Equations 9 and 10 represent two new steps in Path 2.

Path 1:

$$M+H_{2}O\to \mathrm{MOH}+H^{+}+e^{-}$$ 1

$$\mathrm{MOH}\to \mathrm{MO}+H^{+}+e^{-}$$ 2

$$\mathrm{MO}+H_{2}O+e^{-}\to \mathrm{MOOH}+H^{+}+e^{-}$$ 3

$$\mathrm{MOOH}\to M+O_{2}+H^{+}+e^{-}$$ 4

Path 2

$$M+H_{2}O\to \mathrm{MOH}+H^{+}+e^{-}$$ 1

$$\mathrm{MOH}\to \mathrm{MO}+H^{+}+e^{-}$$ 2

$$\mathrm{MO}+H_{2}O+e^{-}\to \mathrm{MH}+H^{+}+O_{2}+e^{-}$$ 9

$$\mathrm{MH}\to M+H^{+}+e^{-}$$ 10

To evaluate the role of electron-rich or electron-deficient states on determining the favorable reaction pathway, we calculated the relative energies of intermediates in both pathways. As previously described, steps 1 and 2 predominantly involve Bi and V active sites, respectively, with intermediates adopting Bi–OH and V–O configurations. To confirm that charge modulation does not alter active site assignments, we computationally validated V–OH and Bi–O structures in charged pristine BiVO_4_ (Table S2 in the SI). Consistently, the V–O configuration exhibited a lower energy than Bi–O in all cases. Additionally, the initial V–OH structures switch to Bi after relaxation in all cases, confirming Bi–OH as the thermodynamically favored intermediate for step 1. Thus, for the first two steps, Paths 1 and 2 are the same.

Figure 5 compares the reaction energies between pristine BiVO_4_ with and without artificial charges across both pathways. Detailed path 2 energetics for all 11 systems are presented in Table S3 in the SI.

**Figure 5:**
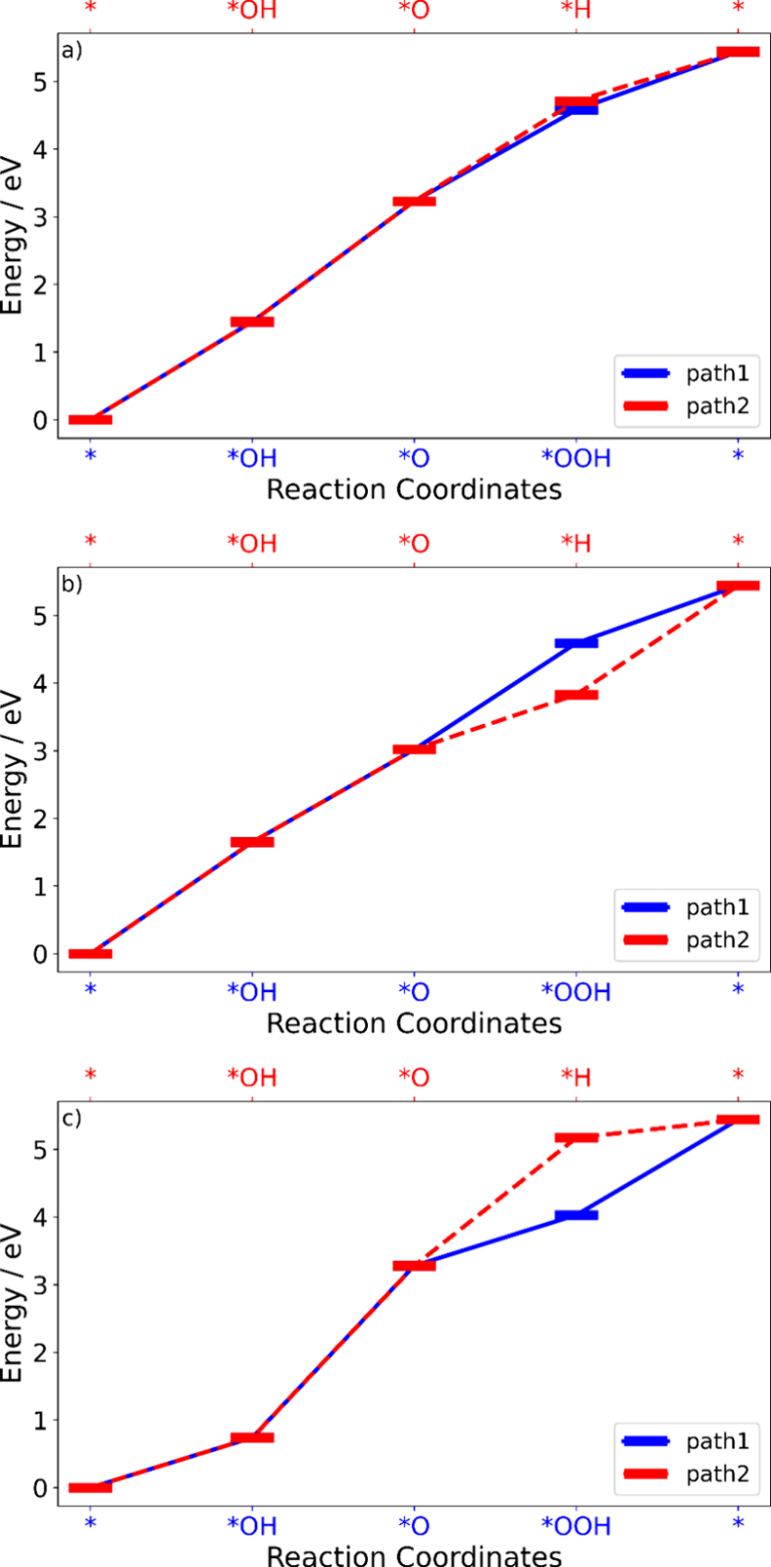
Relative energy of each intermediate of OER on (a) pristine BiVO_4_, (b) election-deficient BiVO_4_, and (c) election-rich BiVO_4_. Equations 3 and 4 are the last two steps in Path 1, while eqs 9 and 10 are those in Path 2.

Distinct variations emerge between the pristine and charged BiVO_4_. For pristine BiVO_4_, step 3 reaction energies are 1.34 eV (Path 1) and 1.48 eV (Path 2), suggesting a negligible preference under neutral conditions. The positive charging drastically reduces the step 3 energy to 0.81 eV (Path 1) versus 1.57 eV (Path 2), promoting early O_2_ generation. Conversely, the negative charge increases the energy of step 3 in Path 2 to 1.90 eV—far exceeding 0.75 eV in Path 1—delaying the release of O_2_ to step 4.

A similar trend is observed in the defect BiVO_4_. For the system with Bi and V vacancies, which are electron-deficient, the step 3 reaction energy is significantly decreased to 0.77 and 0.76 eV, respectively. However, for the O vacancy with electron richness, the step 3 reaction energy is increased to 1.79 eV.

In heterojunction systems, changes may not be so significant but the electron richness/deficiency still has some effect. The BiVO_4_/TiO_2_ heterojunction with no excess charge has a step 3 reaction energy of 1.36 eV. A lower step 3 energy of 1.26 eV was obtained for the BiVO_4_/WO_3_ heterojunction, where electrons were drawn to WO_3_, making BiVO_4_ electron-deficient. Notably, we did not attain a stable intermediate of M–H in the BiVO_4_/ZnO system. The inexistence of such an intermediate may also confirm that the electron richness is not favorable for Path 2. Thus, for all of the systems we considered, the electron-deficient systems have preferences for Path 2, while election-rich systems have preferences for Path 1.

The reason why electron deficiency favors Path 2 correlates with the binding energy of Bi–O. As shown in Table S1 in the SI, the electron deficiency weakened the Bi–O bond. The −OH group from the second H_2_O is also affected by such weakening, resulting in a lower tendency to bond with Bi when forming the M–OOH intermediate in step 3. With a more stable M–H intermediate in electron-deficient systems, the reaction pathway consequently switches to Path 2.

## 4 Conclusions

An extensive DFT study was performed on pristine BiVO_4_, heterojunction systems (BiVO_4_/WO_3_, BiVO_4_/TiO_2_, and BiVO_4_/ZnO), charged BiVO_4_, and BiVO_4_ with atomic defects. Reaction energies were calculated for the steps of the OER process. The results reveal a detailed understanding of the relation between the reaction energies and the role of electron richness/deficiency. Our results show that the electron richness decreases Δ*E*_1_ and Δ*E*_3_, while it increases Δ*E*_2_ and Δ*E*_4_. Electron deficiency plays the opposite role. A linear relationship between the excess charge and reaction energy (Δ*E*_1_ and Δ*E*_2_) was established: Δ*E*_1_ increases while Δ*E*_2_ decreases with more positive charge. Adsorption energies of different species were calculated to investigate how the electron richness and/or deficiency affects the reaction energies. Our results show that the Bi–O bond is weakened in the electron-deficient system and the V–O bond is strengthened. This relationship was confirmed both in charged BiVO_4_ by setting the system a nonzero total charge, and the more realistic model by forming heterojunctions or vacancies on BiVO_4_.

Besides, the study also explores alternative reaction pathways, showing how electron richness or deficiency can shift reaction preferences in O_2_ generation between an earlier or later reaction step. Our results showed that O_2_ prefers to be released at step 3 in electron-deficient systems, while for electron-rich systems, O_2_ prefers to be released at step 4.

This comprehensive analysis highlights the complex interplay among charge, defects, and heterojunctions, contributing to a deeper understanding of how these factors can enhance or modify the performance of the OER on BiVO_4_ surfaces. Our work showed the possibility of tuning the reaction energy through commonly used methods, and then further decreasing the overpotential of the BiVO_4_-based water-splitting catalyst could be achieved.

However, the reason for the anomalies of the O vacancy system remains unknown, and further study is needed to investigate the significant deviation from the predicted Δ*E*_1_. Besides, the adsorption energy of the OH group in all heterojunction systems become smaller compared to that of pristine BiVO_4_ with the electron rich heterojunction having a stronger adsorption than the electron deficient heterojunction. The factors influencing the OER energies in heterojunction systems need further investigation.

## Supplementary Material



## Notes

Structures of BiVO_4_; heterojunction BiVO_4_ and defect BiVO_4_ considered in the study; linear fitting of Δ*E* versus the excess charge with the data of the O_vac_ system included; adsorption energies; reaction energies of Path 2 (PDF)
