# Supplementary material for: Exploring the Effects of Charge Modulation on the Reaction Energetics of the Oxygen Evolution Reaction in BiVO4‑Based Photocatalysts
Source: ACS Omega. 2025 Oct 13;10(41):48167–75. doi: 10.1021/acsomega.5c04810 (PMC12547797; doi:10.1021/acsomega.5c04810)
Supplement: Supplementary file 1 [file ao5c04810_si_001.pdf]

Supporting Information for

Exploring the Effects of Charge Modulation on the Reaction Energetics of the Oxygen Evolution Reaction in $BiVO_4$-Based Photocatalysts

Hongjiang Chen[b], Patrick H.-L. Sit [a,b,*]

[a] School of Energy and Environment, City University of Hong Kong, Tat Chee Avenue, Kowloon, Hong Kong, China

[b] City University of Hong Kong Shenzhen Research Institute, Shenzhen 518057, China

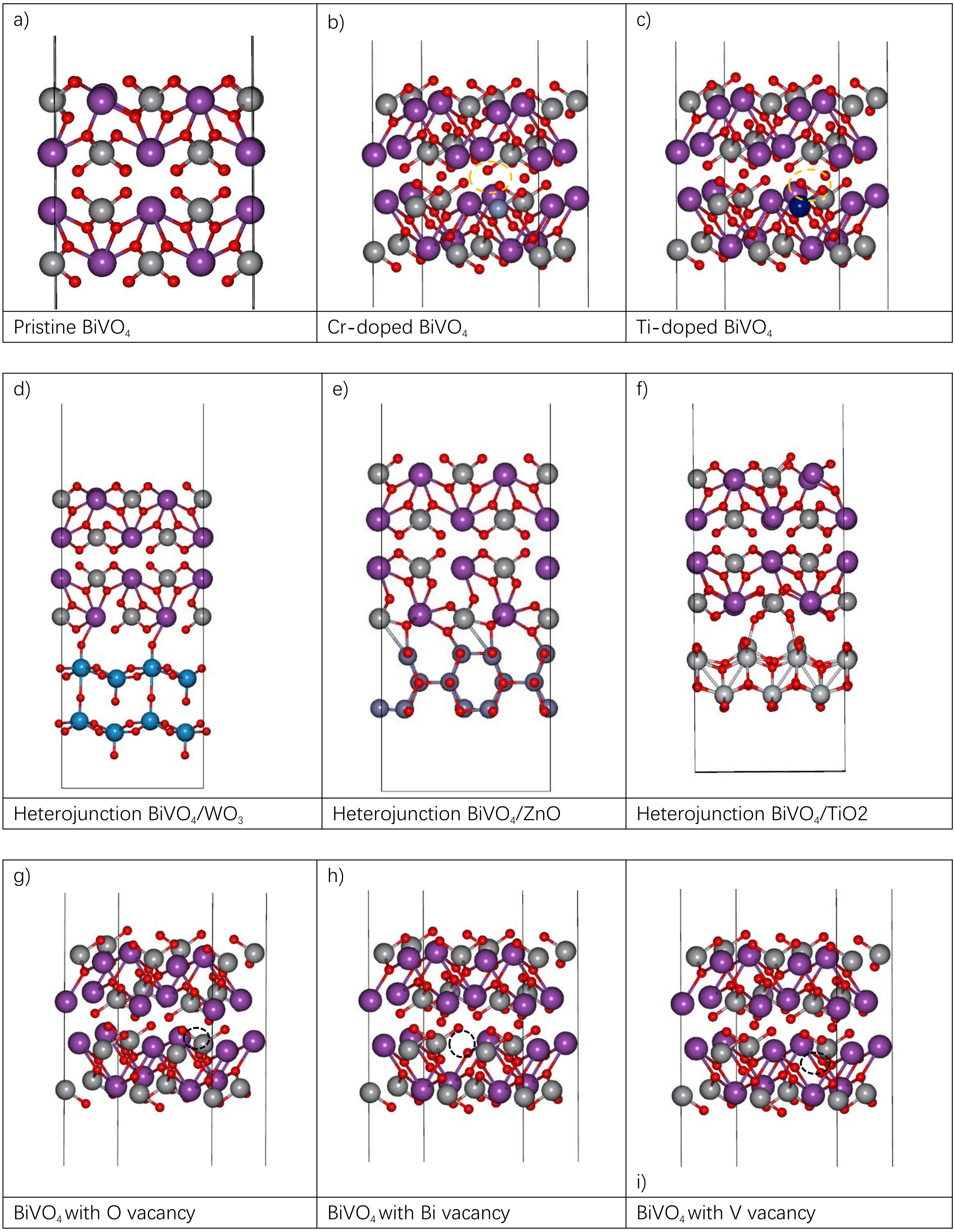

Figure S1. Structures of $BiVO_4$, heterojunction $BiVO_4$ and defect $BiVO_4$ considered in the study. Positions of the dopants and vacancies are labeled in a black dash oval. a) pristine $BiVO_4$; b) and c) doped $BiVO_4$; d), e), f)

heterojunction $BiVO_4$ systems; g), h), i) defect $BiVO_4$

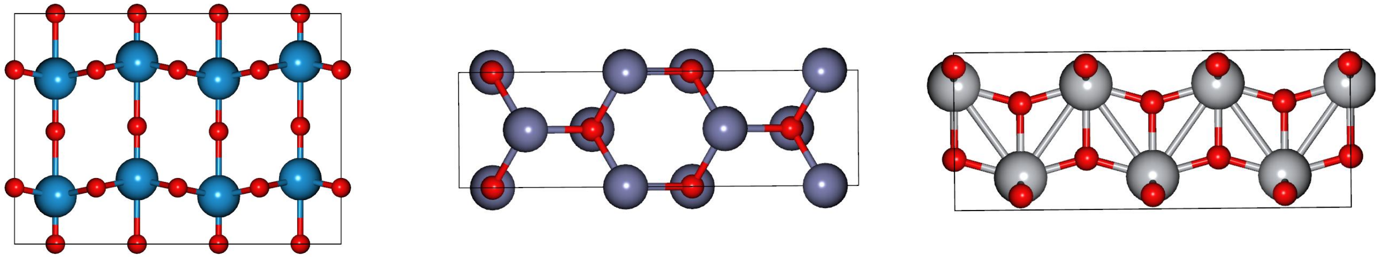

	WO_3	ZnO	TiO_2
Original cell size	5.56 × 5.56 × 3.75, α=β=γ=90°	3.19 × 3.19 × 5.10, α=β= 90°, γ=120°	3.73 × 3.73 × 9.37, α=β=γ=90°
Adjusted cell size	5.09 × 5.19 × 3.75, α=β=γ=90°	3.19 × 3.19 × 5.00,α=β= 90°, γ=120°	3.34 × 3.34 × 9.37, α=β=γ=90°
Slab size *	10.18 × 10.38 × 7.33, α=β=γ=90°	10.00 × 10.37 × 3.19, α=β=γ=90°	10.02 × 10.02 × 5.62, α=β=γ=90°
Facet**	(001)	(110)	(001)
Number of atoms	64	48	54
Mismatch with $BiVO_4$ ***	0.19%	2.68%	4.99%

Figure S2. Structures of the compounds used to build the heterojunction. *Slabs were constructed using the adjusted cell. **The facets that contact with (010) $BiVO_4$. ***Mismatch of the contact surface areas were compared between slab size and the 10.17 × 10.38 × 32.01 Å^3 $BiVO_4$

Discussed structures	Tested structures
a) 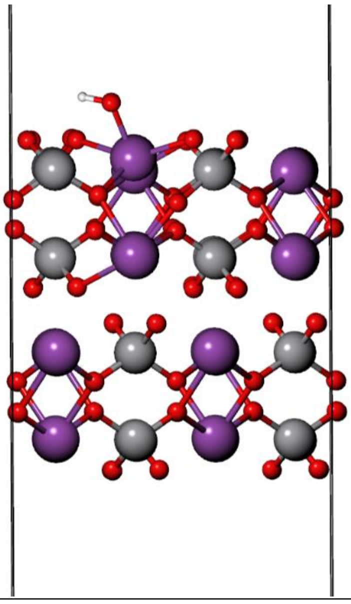	
0	
b) 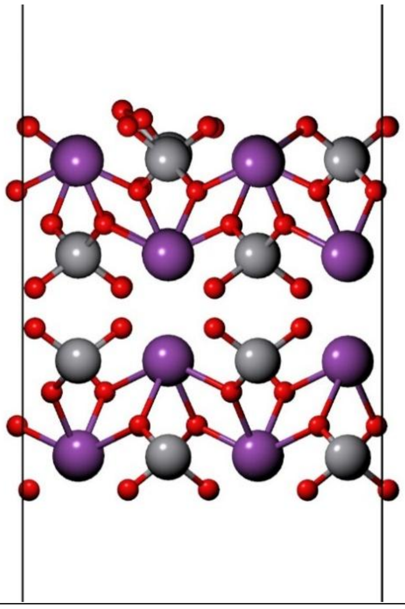	c) 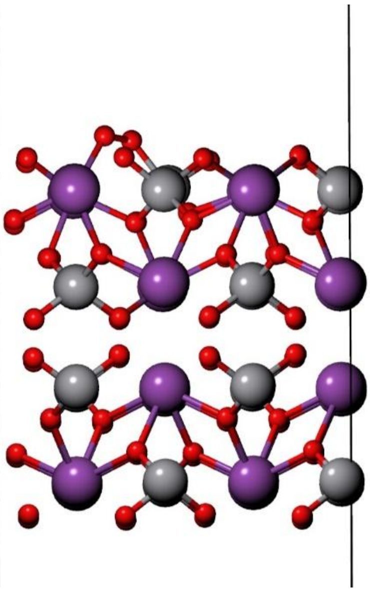
0	0.5 eV

d) 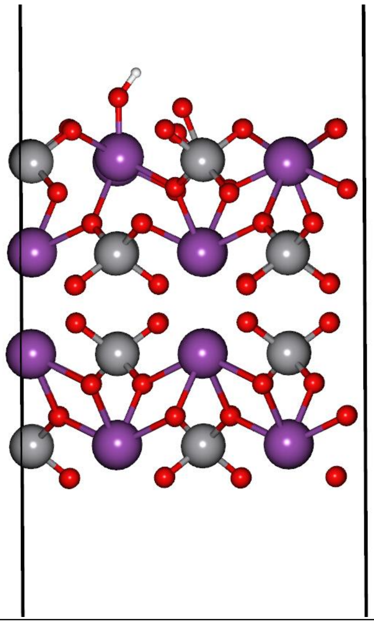	e) 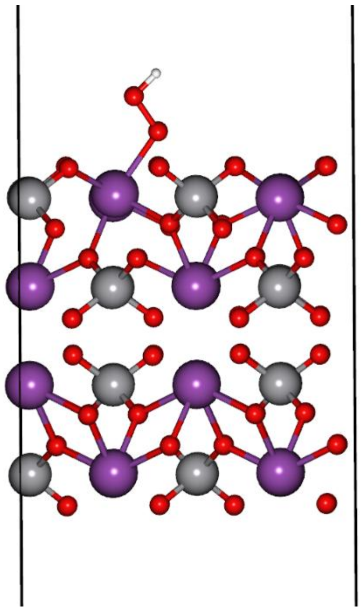
0	-0.03 eV

Figure S3. Discussed structures in main section and tested structures for each intermediate in OER process and their relative energies. a) intermediate M-OH, -OH group binds with Bi; b) intermediate M-O, -O binds with V; c) intermediate M-O, -O binds with Bi; d) intermediate M-OOH, -OH bind with Bi and O bind with V; e) intermediate M-OOH, -OOH group bind with Bi.

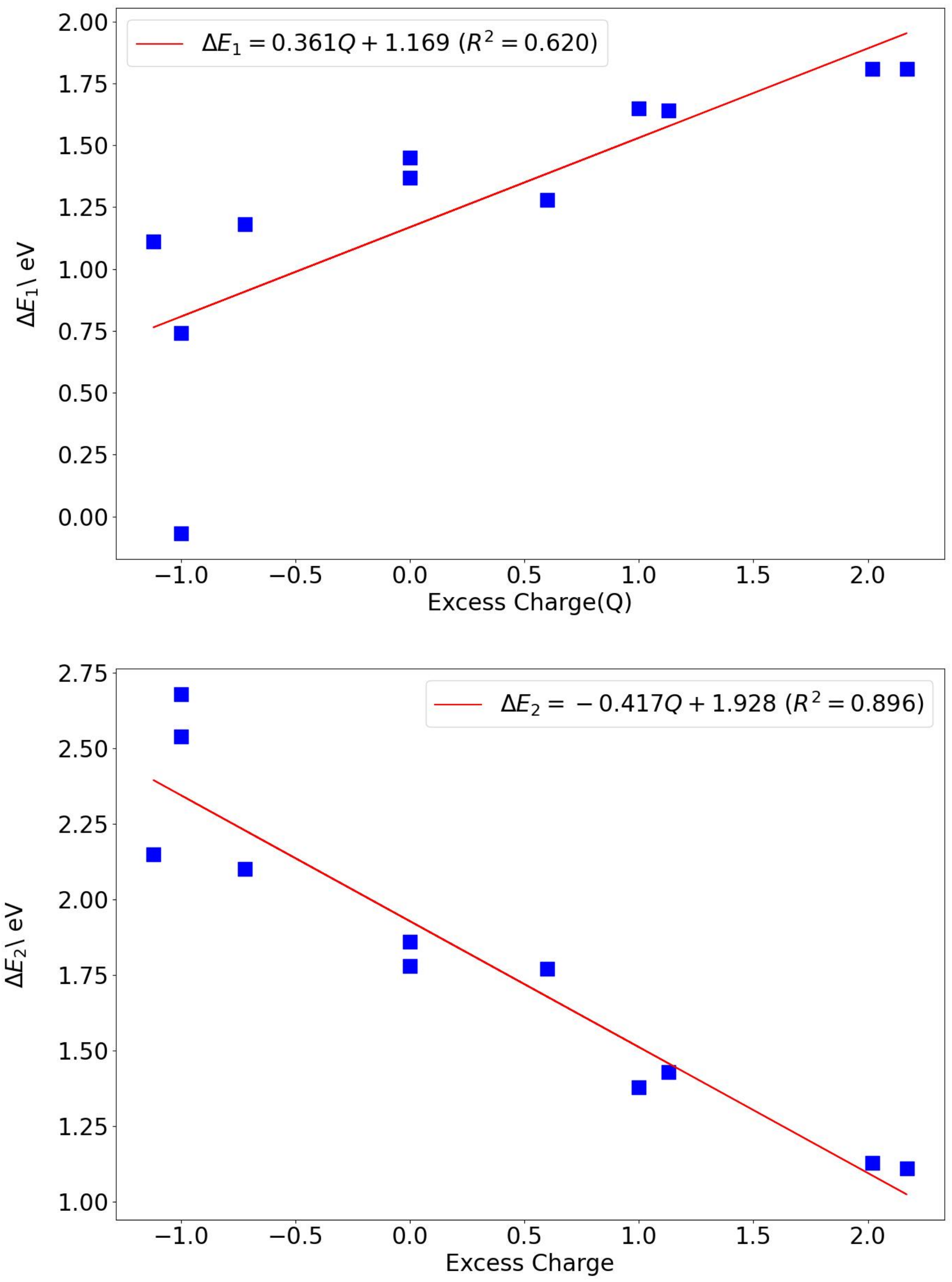

Figure S4. ΔE_1 and ΔE_2 versus the excess charge with the data of the O_{vac} system included in the linear fitting

Table S1. Adsorption energies of -OH and -O on the different system and their differences from those in pristine $BiVO_4$. Negative ΔE_{ad} values mean stronger adsorption(bonding). Unit in eV.

		E_{ad} (-OH)	E_{ad} (-O)	ΔE_{ad} (-OH)	ΔE_{ad} (-O)
Electron Deficiency Systems	+1	-1.52	-3.40	0.20	-0.21
	Ti	-1.53	-3.35	0.19	-0.16
	WO_3	-1.88	-3.37	-0.17	-0.18
	Bi_{vac}	-1.35	-3.47	0.36	-0.29
	V_{vac}	-1.36	-3.50	0.36	-0.32
Neutral Systems	Pristine	-1.72	-3.19	0.00	0.00
	TiO_2	-1.79	-3.19	-0.08	0.00
Electron Richness Systems	-1 charge	-2.43	-3.14	-0.71	0.05
	Cr	-2.06	-3.16	-0.34	0.03
	ZnO	-1.98	-3.13	-0.27	0.05
	O_{vac}	-3.24	-3.81	-1.52	-0.62

Table S2. Relative energies of the intermediates in Step 2 in pristine $BiVO_4$ with or without charge. Only the MO intermediates are listed since -OH group keeps bonding with Bi regardless of the starting configuration.

Excess Charge	1	0	-1
Bi-O	97.22kcal/mol	11.92kcal/mol	6.27kcal/mol
V-O	0	0	0

Table S3. Comparison of the reaction energies of Step 3 and Step 4 between Path 1 and Path 2 on different $BiVO_4$ surfaces.

		Path 1		Path 2	
Excess Charge	System	Step 3	Step4	Step 3	Step 4
`+1.00	Pristine	1.57	0.84	0.81	1.6
+0.60	WO_3	1.34	1.05	1.26	1.12
+1.13	Ti-doped	1.60	0.77	0.99	1.38

+2.02	Bi_{vac}	1.81	0.68	0.77	1.72
+2.17	V_{vac}	1.80	0.72	0.76	1.76
0	Pristine	1.34	0.86	1.48	0.73
0	TiO_2	1.29	0.91	1.36	0.85
-1.07	O_{vac}	0.63	0.72	1.79	1.04
-1.12	Cr-doped	1.08	1.10	1.55	0.63
-0.72	ZnO	1.15	1.00	-	-
-1.00	Pristine	0.75	1.41	1.9	0.26
